# Revisiting motor inhibition in individuals with functional paralysis and spinal cord injury

**DOI:** 10.1093/braincomms/fcaf377

**Published:** 2025-09-27

**Authors:** Vanessa Vallesi, Cécile Galléa, Jothini Sritharan, Elia R Hurni, Inge Eriks-Hoogland, Michaela Gegusch, Johannes Slotboom, Yulia Worbe, Emmanuel Roze, Anke Scheel-Sailer, Rajeev K Verma, Giuseppe A Zito

**Affiliations:** Support Centre for Advanced Neuroimaging (SCAN), Institute for Diagnostic and Interventional Neuroradiology, Inselspital, Bern University Hospital, University of Bern, 3010 Bern, Switzerland; Swiss Paraplegic Research, 6207 Nottwil, Switzerland; Swiss Paraplegic Centre, 6207 Nottwil, Switzerland; Paris Brain Institute, INSERM, CNRS, Sorbonne Université, 75013 Paris, France; Swiss Paraplegic Research, 6207 Nottwil, Switzerland; Swiss Paraplegic Centre, 6207 Nottwil, Switzerland; Faculty of Health Sciences and Medicine, University of Lucerne, 6002 Lucerne, Switzerland; Swiss Paraplegic Research, 6207 Nottwil, Switzerland; Swiss Paraplegic Centre, 6207 Nottwil, Switzerland; Swiss Paraplegic Research, 6207 Nottwil, Switzerland; Swiss Paraplegic Centre, 6207 Nottwil, Switzerland; Faculty of Health Sciences and Medicine, University of Lucerne, 6002 Lucerne, Switzerland; Clinic for Neurology, Cantonal Hospital St Gallen, 9007 St Gallen, Switzerland; Support Centre for Advanced Neuroimaging (SCAN), Institute for Diagnostic and Interventional Neuroradiology, Inselspital, Bern University Hospital, University of Bern, 3010 Bern, Switzerland; Paris Brain Institute, INSERM, CNRS, Sorbonne Université, 75013 Paris, France; Paris Brain Institute, INSERM, CNRS, Sorbonne Université, 75013 Paris, France; Centre for Rehabilitation and Sport Medicine, Inselgroup, Bern University Hospital, 3010 Bern, Switzerland; Swiss Paraplegic Research, 6207 Nottwil, Switzerland; Swiss Paraplegic Centre, 6207 Nottwil, Switzerland; Swiss Paraplegic Research, 6207 Nottwil, Switzerland; Faculty of Health Sciences and Medicine, University of Lucerne, 6002 Lucerne, Switzerland

**Keywords:** functional neurological disorder, spinal cord injury, functional connectivity, go/no-go, task-based fMRI

## Abstract

Functional paralysis (FP), a subtype of functional neurological disorder (FND), is characterized by symptoms of paralysis without clinically evident damage to the nervous system. Previous research has reported impaired inhibitory control in FND, such as weaker inhibition and slower response latencies, yet the underlying neural correlates remain unclear. Moreover, it is unknown whether such neural correlates depend on the symptoms or are a specific trait of the disorder. To address this gap, we compared individuals with chronic FP, healthy controls (HCs) and individuals with spinal cord injury (SCI), who present a similar symptom phenotype but differ in the cause of their condition, hence provided the opportunity to disentangle disorder-specific from symptom-specific patterns of neural activity. In this observational study, 16 patients with FP (6 males/10 females; mean age = 39.4 ± 13.1 years), 24 patients with SCI (18 males/6 females; mean age = 42.4 ± 11.6 years) and 29 HC (8 males/21 females; mean age = 35.5 ± 12.6 years) underwent event-related functional MRI while performing a go/no-go task. We conducted a task-related validation analysis to isolate functional networks involved in response inhibition, and implemented a generalized psychophysiological interaction (gPPI) focusing on the associative motor network. We then compared behavioural performance and patterns of functional connectivity across groups with a general linear model. No group differences in task performance emerged, suggesting intact motor inhibition in FP. Validation analysis over the whole sample showed the recruitment of typical regions of the motor inhibition network. Compared with HC, we identified significant differences in functional connectivity (*P* < 0.05, family-wise error corrected) in both paralysis groups (FP and SCI), which exhibited higher functional connectivity between the right precentral gyrus and the left insula during response inhibition. Functional connectivity during response inhibition was similar between patient groups. These findings suggest a shared neural pattern associated with symptoms of paralysis, rather than a disorder-specific deficit, and may reflect an abnormal limbic drive of the motor network involved in movement initiation.

## Teaser

Vallesi *et al*. report that individuals with functional paralysis and spinal cord injury show similar increases in functional connectivity between the right precentral gyrus and left insula during motor inhibition, despite intact behavioural performance. These findings suggest a shared neural adaptation linked to paralysis symptoms, rather than a disorder-specific deficit.

## Graphical Abstract

**Graphical Abstract:**
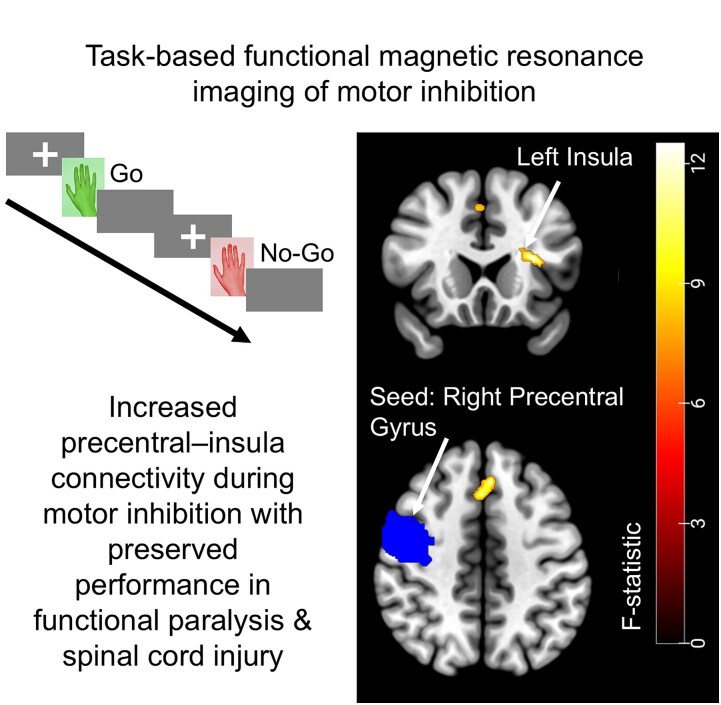

## Introduction

Functional neurological disorder (FND) presents with neurological symptoms without clinically evident damage to the nervous system, although emerging neuroimaging studies suggest potential group-level microstructural brain alterations.^1,2^ The clinical presentation of FND encompasses a wide range of symptoms, including, but not limited to, functional seizures, persistent perceptual postural dizziness, functional cognitive disorder and functional movement disorders.^3^

To date, the underlying pathophysiology of FND is still unknown, and consequently, effective treatments and prognostic indicators are lacking.^4^ The aetiology of FND is considered to involve a complex interplay of biological, psychological and social factors, none of which alone is sufficient to causally explain the disorder.^5-7^ While psychiatric comorbidities, such as depression and anxiety, are frequently observed in FND, they do not appear to directly influence the disorder's outcome.^8^ Moreover, given the heterogeneity among individuals with FND, multiple combinations of predisposing factors may result in similar functional symptoms.^9^

Studies focusing on FND as a whole have documented neurocognitive impairments in multiple domains. For instance, information processing speed is more severely affected in FND than in other somatic disorders.^10^ Additional deficits include spatial working memory and attention^11^ as well as executive functions.^12^ It is assumed that the impaired cognitive integrative functions observed in FND are not directly associated with comorbid symptoms of anxiety or depression but may underlie disorder-specific processes.^13^ Notably, inhibitory control, a subdomain of executive functions, is impaired, with the functional seizure subtype showing greater deficits than genetic generalized epilepsy.^14^ Van Wouwe *et al*.^15^ found that individuals with FND exhibit slower response latencies in both action initiation and action cancellation tasks compared with healthy controls (HC) and make more errors in interference control when responding to conflicting stimuli. Similarly, Hammond-Tooke *et al*.^16^ reported that people with FND demonstrate longer response time and a higher number of errors in motor inhibition tasks using the go/no-go paradigm compared with HC.

One possible explanation for the impaired neurocognitive domain, specifically motor inhibition in FND, is alterations in the serotonergic system. Individuals with FND exhibit changes in their serotonergic pathways, which are associated with clinical outcomes and symptom severity.^17^ Moreover, direct serotonin release is known to influence performance in motor inhibition tasks.^18^

To date, little is known about the brain activity associated with impaired motor inhibition in FND. In a study on functional paralysis (FP), a subtype of FND characterized by motor deficits such as paralysis, the left inferior frontal gyrus was found active during passive movements of the affected hand and impaired inhibitory control was suggested to contribute to symptom production.^19^ However, another case report examined brain activity during motor inhibition using a go/no-go task found abnormal activity in the precuneus, ventromedial prefrontal cortex and left orbitofrontal cortex—regions typically associated with self-related representations rather than inhibitory control. Conversely, the same study reported that a group of HC exhibited activation in the right inferior frontal gyrus and inferior parietal lobule, areas more commonly involved in inhibitory control.^20^ Building upon this initial single-case finding, our research aims to generalize these insights to a broader FP cohort. We investigated patterns of functional connectivity associated with proactive motor inhibitory control in FP using event-related functional MRI (fMRI) during a go/no-go task. Functional connectivity analysis allows for the examination of network-level interactions between brain regions, which is particularly relevant in FP, where large-scale network disruptions are central to the disorder.^21^

According to Bayesian Brain Theory and the Predictive Processing framework, the brain continuously predicts sensory inputs and updates these predictions based on prediction errors—discrepancies between expected and actual sensory experiences. This process facilitates efficient energy regulation (allostasis).^22^ In FND, chronic prediction errors may lead to a brain state of ‘fatigue’ or ‘hyperarousal’ as the brain attempts to maintain allostasis despite a constant mismatch, resulting in energy mismanagement.^23^ In FP, we hypothesize that these chronic mismatches extend beyond sensory processing to motor control, disrupting large-scale networks involved in motor inhibition.

The novelty of our study is that, in order to determine whether motor inhibition and its neural correlates in FP are due to the underlying disorder processes, we included two comparison groups: individuals with spinal cord injury (SCI) exhibiting similar motor symptoms and a second control group of healthy participants. In SCI, paralysis arises from disruptions in afferent and efferent sensorimotor pathways,^24^ whereas in FP, such disruptions are not identifiable. Despite this difference, both conditions present a similar phenotypic manifestation of motor deficits. Furthermore, individuals with SCI are affected by neurocognitive impairment, with traumatic brain injury, psychiatric disorders, medication side effects and pain being contributing factors.^25^ Of note, individuals with SCI show impaired executive functions, such as verbal fluency,^26^ but not in the specific domain of inhibitory control.

FP and SCI share phenotypically similar motor impairments but differ in their neurobiological mechanisms. Investigating motor-related regions within the inhibitory control network^27^ allows us to differentiate symptom-related alterations (neural changes resulting from the presence of paralysis, regardless of aetiology) from condition-specific changes. We focused on motor-associated regions, such as the precentral gyrus and supplementary motor area, as these areas may reflect disorder- or symptom-specific connectivity changes.

If motor inhibition alterations in FP arise from central dysfunction (energy mismanagement), we would expect FP-specific connectivity changes in motor-inhibitory regions, distinct from SCI, where motor impairments stem from peripheral neuronal disruption. Conversely, overlapping alterations in both groups would suggest symptom-related effects.

## Materials and methods

### Design and ethical approval

This study employed a cross-sectional design. The reporting of this study was conducted according to the STROBE guidelines. Ethical approval was obtained from the local Ethics Committee of Northwest and Central Switzerland (approval number: 2021-01775). All participants provided written informed consent in accordance with the Declaration of Helsinki. The study was preregistered on ClinicalTrials.gov (ID: NCT05139732).

### Participants

The study was conducted at the Swiss Paraplegic Centre in Nottwil, Switzerland. Participants were primarily recruited through the Swiss Paraplegic Centre via online and in-clinic flyers, as well as physician referrals. Additional participants came from the Clinic for Neurology at the Cantonal Hospital St Gallen. The sample size of 74 was determined a priori using G*Power^28^ (see Supplementary material for parameter details). The total initial sample size consisted of 71 participants, with 2 participants excluded due to excessive motion during fMRI scans, resulting in a final sample size of 69. The FP group (*n* = 16, 6 males/10 females, mean age = 39.4 ± 13.1 years) was age-matched with HC (*n* = 29, 8 males/21 females, mean age = 35.5 ± 12.6 years). The SCI group consisted of 24 participants (18 males/6 females, mean age = 42.4 ± 11.6 years). Detailed demographic information is provided in Supplementary Tables 1 and 2. The inclusion criteria were: (i) having symptoms for at least 6 months (for all patients); (ii) being able to hold a pen (for all patients) and (iii) age between 18 and 60 years. The exclusion criteria were: (i) having contraindications for magnetic resonance imaging; (ii) having a history of neurological disorders (except for FND and SCI for the respective patient groups); (iii) having any acute psychological disorder and (iv) having red-green colour vision deficiency.

### Psychometric and functional assessment tools

The Hospital Anxiety and Depression Scale (HADS) was used to assess anxiety and depression levels among the participants. This includes two subscales: one for anxiety (HADS-A) and one for depression (HADS-D).^29^ Pain intensity during the measurement and over the past 7 days was measured using the Numeric Rating Scale.^30^ To evaluate quality of life, the Satisfaction with Life Scale (SWLS) was administered.^31^ Additionally, all patients completed the Spinal Cord Independence Measure (SCIM) to assess their functional independence.^32^ The International Standards for Neurological Classification of Spinal Cord Injury (ISNCSCI)^33^ was performed by certified physicians to determine the severity of paralysis in both FP and SCI groups (Supplementary Table 1).

### Go/no-go task

**Figure 1:**
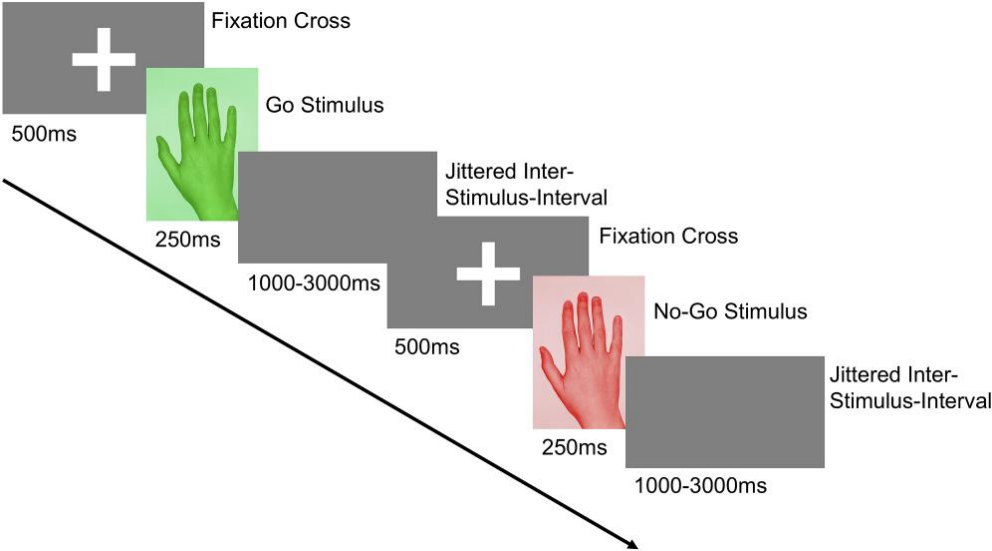
**Go/no-go task design.** In this inhibitory control task, each trial began with a fixation cross, followed by the pseudo-random presentation of either a go-stimulus or a no-go stimulus. After the stimulus, a blank screen with a jittered interstimulus interval of 1000 to 3000 ms was displayed.

All participants completed an event-related go/no-go task during fMRI scanning using their dominant hand. The task, adapted from Cojan *et al*.^20^ and implemented in PsychoPy,^34^ is detailed in Fig. 1. Participants were instructed to respond as quickly as possible by pressing a button on an MR-safe device when presented with a go signal, and to withhold their response when a no-go signal appeared. Response times and accuracy were recorded for each trial. Response times were analysed only for correct go and no-go trials, and outliers (defined as response times >2 standard deviations from the mean) were excluded from the analysis.^35^ Overall task accuracy was calculated for each participant as the percentage of correct responses (correct trials/total trials × 100), where correct trials included both hits (correct responses to go trials) and correct rejections (withheld responses on no-go trials). These individual accuracy percentages were then averaged across groups for statistical comparisons. Detailed accuracy analyses for each condition are reported in the Supplementary material, including go trial accuracy (hit rate: hits/[hits + misses] × 100) and no-go trial accuracy (correct rejection rate: correct rejections/[correct rejections + false alarms] × 100).

Following eight practice trials inside the scanner, participants completed four blocks of 52 trials each. To maintain consistent motivation, performance feedback, expressed as a percentage of correct responses, was provided after each block. Stimuli were presented in a pseudorandom order, with go stimuli comprising 75% of trials and no-go stimuli 25%, ensuring the unpredictability of the no-go stimuli.^20^ This ratio produces the highest error rates,^36^ maximizing sensitivity for detecting inhibitory control deficits.

### Brain imaging acquisition and pre-processing

Neuroimaging data were collected using a 3 T MRI scanner (Philips Achieva, release 5.4.1; Philips Healthcare, Best, The Netherlands) equipped with a 32-channel head coil (Philips Healthcare). Participants were positioned supine within the scanner with an MR-safe response device placed in their dominant hand. The MR imaging protocol included anatomical scans, and task-based fMRI sequences, with the entire procedure lasting ∼20 min (for acquisition parameters, see Supplementary material). Functional and anatomical data were pre-processed using the CONN Toolbox, version 22a,^37^ including realignment with correction of susceptibility distortion interactions, outlier detection, direct segmentation and MNI-space normalization and smoothing. Excessive motion during fMRI was defined as mean motion estimates exceeding the third quartile plus three times the interquartile range (Q3 + 3IQR) or falling below the first quartile minus three times the interquartile range (Q1–3IQR).^38^ Participants exceeding these thresholds were excluded from further analysis. The detailed pre-processing steps are listed in the Supplementary material.

### Functional connectivity and statistical analysis

To ensure that participants remained focused throughout the task-based fMRI session, we performed control measurements on behavioural performance. We examined participants’ response time across the entire time series and assessed accuracy within each of the four task blocks. Behavioural data were analysed using non-parametric methods due to violations of parametric model assumptions (see Supplementary material). Mean response time and accuracy in percentages were calculated for each participant, and group differences were tested using the Kruskal–Wallis test.

For the imaging data, the conditions of correct rejection versus hit, were analysed to isolate inhibition-specific neural activity, controlling for shared visual processing.^39^ To ensure the validity of the go/no-go task for the functional connectivity analysis, we assessed activation patterns during motor inhibition (correct rejection versus hit) and motor execution (hit versus correct rejection) across the entire sample. This validation was conducted via a voxel-wise analysis using a flexible factorial design in the Statistical Parametric Mapping software (SPM12) running under MATLAB R2021a (The MathWorks, Inc., Natick, MA, USA). ****The model included two factors: (1) group and (2) condition (hit and correct rejection), as well as their interaction. This allowed us to identify the networks involved and verify whether the task elicited the expected activation patterns, confirming its successful implementation. Contrasts of interest compared activation between groups and across conditions, thresholded at *P* < 0.001 (voxel-level) with *P* < 0.05 family-wise error (FWE) correction at the cluster level.

To study changes in functional connectivity (FC) across these conditions, a generalized psychophysiological interaction (gPPI) analysis was used.^40^ The Automated Anatomical Labelling Atlas 3 (AAL3)^41^ was used for all neuroimaging analyses. Based on the meta-analyses of the go/no-go task by Zhang et al.,^27^ we selected motor-associated regions of interest (ROIs) that are implicated in motor inhibition. Specifically, we tested three seeds, i.e. the right and left precentral gyrus, which have been linked to the execution and control of voluntary movements,^42^ and the right supplementary motor area, which is known to play a crucial role in motor planning and timing of inhibitory processes.^43^ The seed blood-oxygen-level-dependent (BOLD) signals were considered the physiological term, whereas the time course of the conditions was considered the psychological term. The multiplication of these two factors formed the psychophysiological interaction term, and functional connectivity changes were characterized using Fisher-transformed semipartial correlation coefficients, providing an estimate of connectivity changes across conditions.

A separate General Linear Model (GLM) was calculated for each voxel,^44^ where the dependent variables were the connectivity measures obtained from the gPPI analysis. For each subject, a connectivity value corresponding to each task condition (hit and correct rejection) was estimated at every voxel. A random-effects analysis was performed at the group level using an analysis of covariance (ANCOVA), with age and handedness as covariates.

Using the Gaussian Random Field theory,^45^ only cluster-level results with FWE correction and a significance threshold of p_FWE_ < 0.05 were considered, thereby minimizing the Type I error (alpha error). Post-hoc group comparisons were then performed with a Bonferroni correction and a significance level of *P* < 0.05. Confidence intervals (CI) of 95% and generalized eta-squared (η^2^_G_) effect sizes were calculated for all significant results. Additionally, η²G was reported for non-significant results to provide context for observed effect sizes.

To assess the robustness of our primary findings, all significant FC and behavioural models were re-estimated with additional covariates known to influence both neural and behavioural measures, namely sex, anxiety/depression scores (HADS-A and HADS-D scores) and medication intake.

In order to verify the association between behavioural performance and neural activity, the Spearman correlation coefficient was calculated between the accuracy on the go/no-go task and patterns of functional connectivity in the clusters showing a significant difference among the three groups. The bootstrap method (10’000 bootstrap samples) was employed to study the robustness of the correlation. To further assess potential confounds, we examined the relationship between time since symptom onset and functional connectivity in both paralysis groups (FP and SCI) separately.

All analyses were conducted using the CONN Toolbox, version 22a^37^ and R software,^46^ with the packages emmeans^47^ and tidyverse.^48^

## Results

### Patient characteristics

There were no significant differences in age across the groups, and no statistically significant association was found for handedness distribution. However, a significant association was found between sex and education among the groups. No significant differences were found between the FP and SCI groups in terms of functional independence, as measured by the SCIM. Similarly, symptom severity, assessed using the ISNCSCI, was comparable between the two patient groups, indicating similar severity of the sensorimotor symptoms.

For HADS-A, 56 out of 69 participants scored within the normal range, 6 (n = 2 FP, n = 2 SCI, n = 2 HC) scored in the mild anxiety range, and 7 (n = 6 FP, n = 1 HC) scored in the severe range, and a significant group difference was observed. For HADS-D, 59 out of 69 participants were in the normal range, 5 (n = 2 FP, n = 3 SCI) were in the mild range and 5 (n = 4 FP, n = 1 SCI) in the severe range, with significant group differences.

On average, participants reported no or mild pain during the experiment. However, the FP group reported moderate pain over the past seven days, which showed a statistically significant difference between the groups. A significant group difference was also found in the SWLS, with the FP group being less satisfied than the SCI and HC groups (Supplementary Table 1). Supplementary Table 2 shows the post-hoc comparisons of demographic and clinical characteristics between the HC, FP and SCI groups.

### Behavioural performance

**Figure 2:**
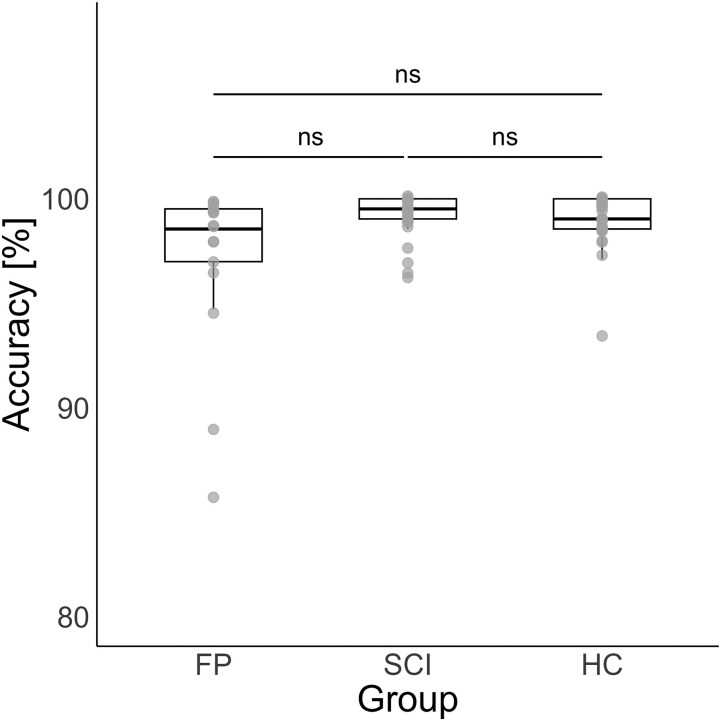
**Task accuracy across groups.** Overall accuracy during the go/no-go task is shown as a percentage (total n = 69). Data were analysed using Kruskal-Wallis test, revealing no significant group differences between individuals with FP, with spinal cord injury (SCI) and HCs (χ2 = 4.33, df = 2, *P* = 0.115, η²_G_ = 0.06). Note that the y-axis is zoomed in for clarity. Individual data points represent participant-level accuracy. Abbreviations: ns = not significant.

Across the four blocks of the go/no-go task, we observed a maximum change of 10 ms in mean response time and a maximum change of 0.8% in accuracy (averaged across participants per block). These small changes suggest that performance was largely consistent across blocks, with only minimal variation (see Supplementary Fig. 1). In the go/no-go task, accuracy showed no significant group difference (see Fig. 2) (χ2 = 4.33, df = 2, *P* = 0.115, η²_G_ = 0.06) with the following distribution per group: FP group (median = 98.6%, interquartile range = 2.52%); SCI group (median = 99.0%, interquartile range = 1.44%); HCs (median = 99.5%, interquartile range = 0.96%). The variability across groups was further examined using Levene’s test, which indicated significant differences in variance across groups (see Supplementary material).

**Figure 3:**
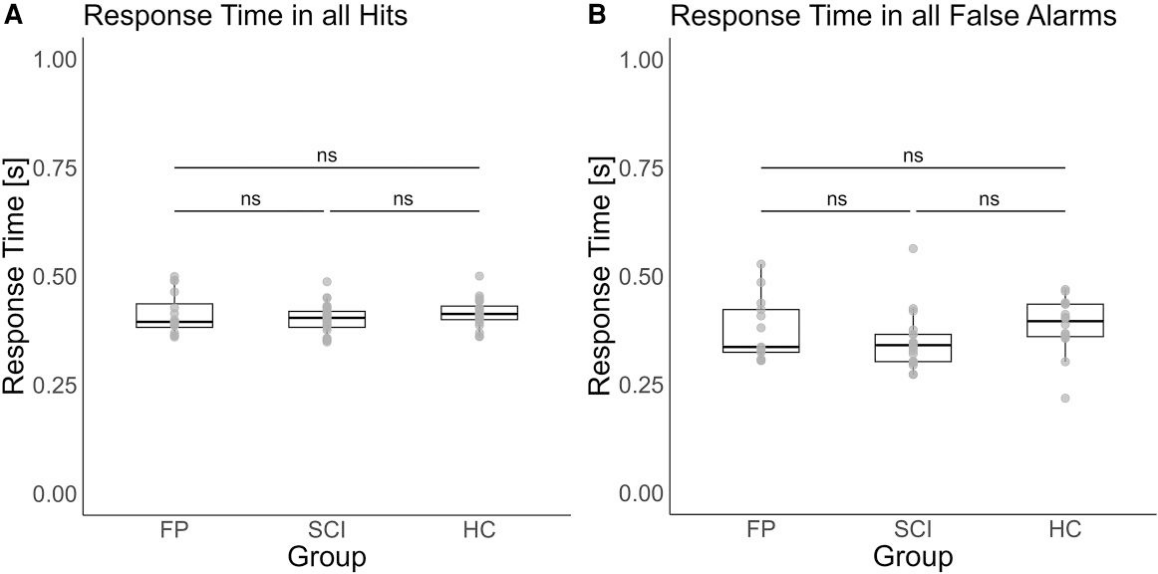
**Response times across groups.** A Kruskal-Wallis test was conducted to examine group differences in response time during the go/no-go task. Individual data points represent participant-level mean response time. (**A**) There were no significant group differences in response time during correct trials (hits) between the FP, spinal cord injury (SCI) group and HCs (χ2 = 2.71, df = 2, *P* = 0.258, η²_G_ = 0.04) (total n = 69). (**B**) There were no significant group differences in incorrect responses (false alarms) between the FP group, SCI group and HC (χ2 = 5.74, df = 2, *P* = 0.057, η²_G_ = 0.12) (total n = 48). Abbreviations: ns = not significant.

The response time for hits did not significantly differ between groups (see Fig. 3A) (χ2 = 2.71, df = 2, *P* = 0.258, η²_G_ = 0.04) with the following response time per group: FP group (median = 0.40 s, interquartile range = 0.10 s); SCI group (median = 0.41 s, interquartile range = 0.07 s); HCs (median = 0.40 s, interquartile range = 0.07 s). The variability in response time between groups was further tested with the coefficient of variation, which showed no difference in variation between groups (see Supplementary material). The model analysing incorrect response time (false alarms) included only a small fraction of the total trials (140 out of 14 352) since accuracy was high and showed no significant group differences (see Fig. 3B) (χ2 = 5.74, df = 2, *P* = 0.057, η²_G_ = 0.12).

### Functional networks of motor inhibition

The validation analysis confirmed activation in the motor inhibitory network across the whole sample (i.e. both patients’ groups and HC together) (see Supplementary Fig. 2 for motor inhibition and Supplementary Fig. 3 for the separate effects of hit and correct rejection).

Voxel-wise comparisons of BOLD activity during correct rejection versus hit trials revealed significant group differences in task-related activation. The flexible factorial analysis identified greater activation in the FP group compared with HC in the left insula and left superior temporal gyrus (Supplementary Table 3). The FP group also showed increased activation relative to HC in the left posterior cingulate cortex and bilateral precuneus. For the contrast HC > FP, significant clusters were located in the left postcentral gyrus and left inferior parietal lobule. Comparisons involving the SCI group demonstrated increased activation relative to both FP and HC in the right postcentral gyrus and right precentral gyrus. The SCI > HC contrast additionally revealed significant activation differences in the bilateral precuneus. Complete statistical results are provided in Supplementary Table 3.

**Figure 4:**
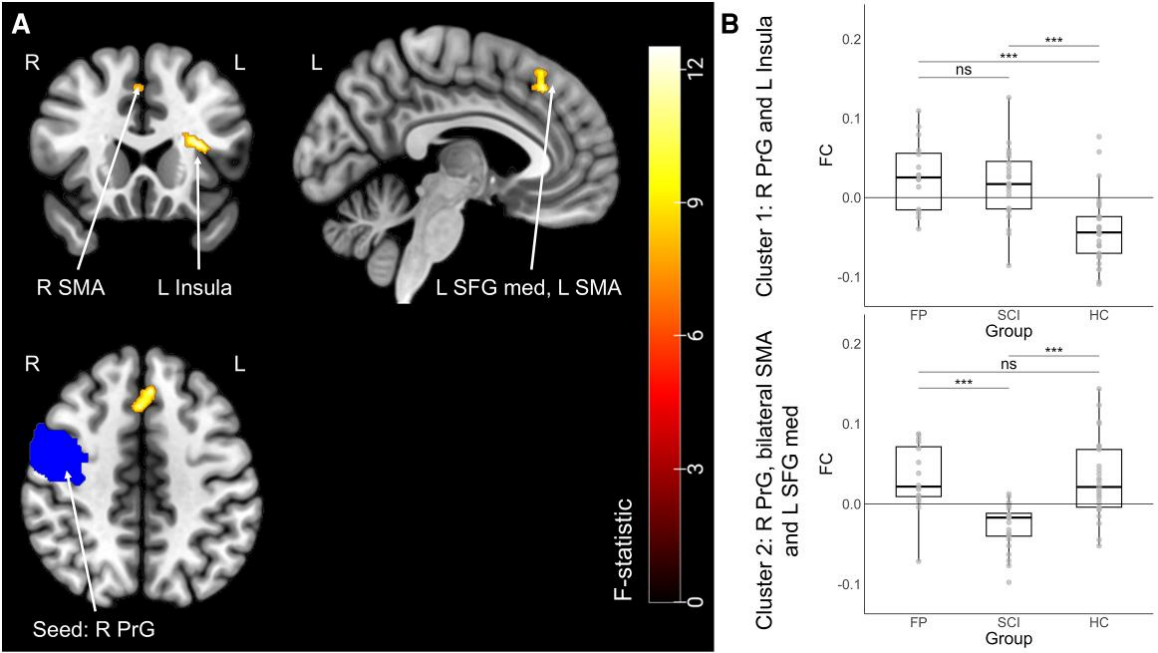
**Group differences in motor inhibition-related functional connectivity.** (**A**) Functional connectivity differences between groups during motor inhibition (using generalized psychophysiological interaction analysis) were observed (individuals with FP: n = 16, individuals with spinal cord injury (SCI): n = 24, HCs: n = 29, total n = 69). (**B**) The right precentral gyrus (R PrG) seed showed significant group differences in functional connectivity (FC) with two clusters. The first cluster included the left insula (F_(2, 64)_ = 12.92, family-wise error-corrected (FWE) *P* = 0.02, η^2^_G_ = 0.35, cluster size = 92 voxels), and the second cluster included the left medial superior frontal gyrus (L SFG med) and the bilateral supplementary motor area (SMA) (F_(2, 64)_ = 19.84, p_FWE_ = 0.03, η^2^_G_ = 0.32, cluster size = 85 voxels). In cluster 1, the FP group and the SCI group had significantly higher FC than HC. In cluster 2, only the SCI group had significantly lower FC compared with both the FP group and HC. Individual data points represent each participant's mean FC value extracted from the respective cluster. Abbreviations: R, right; L, left; SMA, supplementary motor area; SFG med, medial superior frontal gyrus; PrG, precentral gyrus; FC, functional connectivity; FP, functional paralysis group; SCI, spinal cord injury group; HC, healthy controls; ns, not significant; *** *P* < 0.001.

Out of the three tested seeds (i.e, right supplementary motor area, right and left precentral gyrus) with the gPPI, one showed significant alterations in functional connectivity among the groups. The FC of the right precentral gyrus (seed) and left insula (cluster 1: F_(2, 64)_ = 12.92, p_FWE_ = 0.02, η^2^_G_ = 0.35, cluster size = 92 voxels), as well as the left medial superior frontal gyrus and the bilateral supplementary motor area (cluster 2: F_(2, 64)_ = 19.84, p_FWE_ = 0.03, η^2^_G_ = 0.32, cluster size = 85 voxels) differed among the groups (Fig. 4 and Table 1). Bonferroni-corrected post-hoc tests revealed that, in the first cluster, the FP group demonstrated significantly higher functional connectivity compared with HC, as did the SCI group compared with the HC. There was no significant difference between the two patient groups. Detailed statistical results are shown in Table 1. In the second cluster, post-hoc tests showed that the SCI group had significantly lower functional connectivity compared with the HC and to the FP group. The FP group showed no significant difference to HC (see Table 1).

**Table 1:** Functional connectivity differences using the right precentral gyrus as seed

Brain Region(s)	Peak MNI	k (voxels)	F-value_(2, 64)_	*P*-value_FWE_	FP versus HC	FP versus SCI	SCI versus HC
Cluster 1							
Left insula	−32 20 14	92	12.92	0.02	5.10***	0.48^ns^	4.93***
					[0.04, 0.11]	[−0.03, 0.04]	[0.03, 0.10]
Cluster 2							
Left medial superior frontal gyrus, bilateral supplementary motor area	−04 30 44	85	19.84	0.03	−0.04^ns^	−4.49***	−5.01***
					[−0.03, 0.03]	[−0.09, −0.03]	[−0.09, −0.03]

Abbreviations: FP, functional paralysis group; HC, healthy controls; SCI, spinal cord injury group; ***, *P* < 0.001; ns, not significant; FWE, family-wise error; MNI, Montreal Neurological Institute.

Note: All *P*-values are family-wise error corrected for 0.05, with a 95% confidence interval and post-hoc tests are adjusted using Bonferroni.

Sensitivity analyses confirmed the robustness of the behavioural and functional connectivity findings when including covariates (Supplementary Table 4).

**Figure 5:**
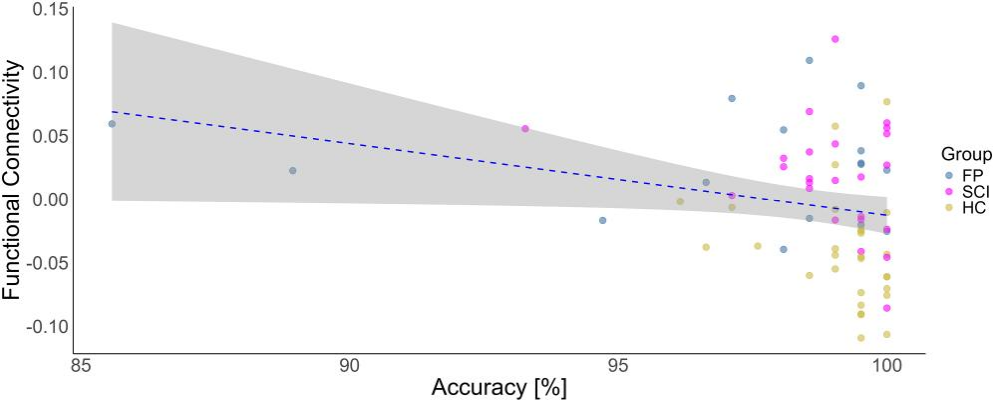
**Precentral gyrus-insula connectivity negatively correlates with inhibitory accuracy.** Spearman correlation between the functional connectivity of the right precentral gyrus and the left insula (cluster 1) with overall accuracy in the whole sample (n = 69, ρ = −0.31, *P* = 0.009). Each data point represents a single participant. Sensitivity analysis excluding two low-accuracy outliers yielded an attenuated but persistent effect (n = 67, ρ = −0.285, *P* = 0.019). Abbreviations: FP, functional paralysis group; SCI, spinal cord injury group; HC, healthy controls.

The correlation analysis revealed that functional connectivity in cluster 1 (ρ = −0.31, *P* = 0.009), but not cluster 2 (ρ = −0.06, *P* = 0.614), significantly correlated with accuracy on the go/no-go task across the entire sample (see Fig. 5). Bootstrapped Spearman correlation analysis confirmed the robustness of this relationship, with a 95% bias-corrected and accelerated confidence interval of [−0.51, −0.06]. The small bias (0.005) and standard error (0.113) further support the reliability of the correlation estimate. When excluding two low-accuracy outliers, the correlation remained significant with ρ = −0.285 and *P* = 0.019. Time since symptom onset was not significantly associated with functional connectivity (cluster 1) in either the FP group (ρ = −0.24, *P* = 0.380) or the SCI group (ρ = 0.03, *P* = 0.905).

## Discussion

In this task-based fMRI study on motor inhibition, no group differences were observed in accuracy on the go/no-go task, nor in response time during hits and false alarms, contrary to our hypothesis. In contrast, functional connectivity during motor inhibition revealed significant group differences. Both the FP and SCI groups exhibited similar connectivity patterns between the right precentral gyrus and the left insula, with both patient groups showing higher connectivity in these regions compared with HC. Notably, connectivity values in this network significantly correlated with accuracy across the whole sample, including FP, SCI and HC groups, indicating that performance during motor inhibition is associated with communication within this functional network. In addition, the SCI group demonstrated lower functional connectivity between the right precentral gyrus, the left medial superior frontal gyrus and the bilateral supplementary motor area, in contrast to the FP group and the HC. This finding was contrary to our hypothesis. These connectivity values were not correlated with accuracy.

While some gPPI findings co-localized with activation differences (notably in FP's left insula and SCI's precentral seed, see Supplementary Table 3), most connectivity effects occurred independently of local BOLD changes. This suggests both region-specific coupling between activation and connectivity, and network-level reorganization distinct from local processing.

Contrary to our findings, prior behavioural studies investigating the go/no-go task in FND have reported reduced accuracy.^49,50^ Hammond-Tooke *et al*.^16^ observed longer response time, with around 15% higher false alarms compared with HC. One possible explanation for the discrepancy in behavioural findings is that previous studies on motor inhibition in FND often focused on positive motor symptoms, such as functional tremor and dystonia,^49^ or mixed subtypes of FND, including functional weakness and co-existing functional seizures.^15,16,50^ In contrast, this is the first study to focus on a homogeneous subtype of FND characterised solely by symptoms of paralysis, in comparison with a population exhibiting similar motor symptoms, such as SCI. Furthermore, in our study, the hand used for the task was unaffected for all participants, excluding the possibility of reduced performance due to a mere motor impairment. It is then possible that previous findings on impaired motor inhibition may have been influenced by the inclusion of participants with positive motor symptoms. These symptoms could have introduced additional complexity, potentially affecting the interpretation of motor inhibition deficits in FND. Because FND encompasses a broad spectrum of behavioural differences, it is essential to consider these variations in behavioural assessments—particularly those relying on response times, such as in our study. Indeed, a recent meta-analysis reported inconsistent results regarding the impairment of neurocognitive functions in FND across various studies and phenotypes.^51^ Acknowledging distinct FND subtypes in both behavioural and neuroimaging research is therefore crucial for deepening the understanding of the mechanisms underpinning FND as a whole, as well as the unique features of each subtype.

Using a version of the go/no-go task similar to that employed by Cojan *et al*.,^20^ our FP group demonstrated overall accuracy (∼ 97% correct responses) comparable to the HC in that study. Their response time (∼ 0.4 s) was also in line with typical measurements, thereby indicating normal task performance across the FP, SCI and HC groups. In fact, response times of around 0.4–0.6 s are generally considered standard in these types of behavioural assessments.^52,53^

Both FP and SCI groups in our study displayed altered functional connectivity between the right precentral gyrus and the left insula during motor inhibition (FC cluster 1). This network is associated with inhibitory control. According to Hoffstaedter,^54^ the premotor cortex is part of the ‘what-network,’ which governs movement selection, whereas the insular cortex is part of the ‘when-network,’ which governs movement timing. The anterior insula is described as a gatekeeper in executive control because it integrates both internal and external multisensory stimuli.^55^ These regions have frequently been discussed in the broader context of FND. For instance, reduced left anterior insular volume has been reported in individuals with FND who experience severe physical health impairments, compared with HC.^56^ In addition, both sensorimotor and insular cortices have been identified as neural correlates of FND in a meta-analysis,^21^ and they have emerged as classifiers for FND in large-scale cross-validation studies involving mixed FND samples.^57,58^ Although we found no significant association between these FC alterations and time since symptom onset, the shared network changes in both paralysis groups could reflect a common neural adaptation—potentially arising in the chronic stage of paralysis. Interestingly, the connectivity between the precentral gyrus and the supplementary motor area (FC cluster 2) showed significant decreases in SCI, compared with FP and HC, consistent with known motor-network alterations in SCI^59^ and potentially reflecting specific disruptions in afferent and efferent spinal–motor pathways unique to SCI.

Within a predictive-processing framework, the insula’s interoceptive and salience functions imply that heightened precentral–insula coupling may reflect increased precision-weighting of motor predictions.^22,23^ In SCI, this could arise from persistent mismatches between intended movement and disrupted sensory feedback (deafferentation), driving compensatory network reorganization. In FP, chronic prediction errors likely stem from maladaptive priors or aberrant attentional focus on bodily signals—though the precise origin of these mismatches remains unknown. Crucially, our data do not allow us to distinguish whether the same connectivity change reflects adaptive plasticity in SCI versus maladaptive processes in FP.

### Limitations and future directions

One limitation of this study is the high accuracy across all groups on the go/no-go task, indicating a possible ceiling effect. While a more demanding inhibition paradigm may be required to reveal subtler deficits, simple go/no-go designs reliably detect gross motor-inhibition impairments in other patient populations (e.g. Parkinson’s disease^60^). The absence of any group differences here thus suggests that large-scale inhibition deficits are unlikely in our FP sample, though finer-grained paradigms may uncover more nuanced alterations. Interpretation of the false-alarm response time model should be approached with caution, as only a small fraction of trials were included due to the high overall accuracy.

Future studies might also consider different phases of the disorder—for example, by comparing subacute and chronic phases—to capture how these conditions evolve over time.

Another limitation of this study is that the FP group exhibited higher mean scores of depression and anxiety compared with both the SCI and HC groups. However, these scores remained in the subclinical range and did not translate into measurable performance deficits: behavioural accuracy and response times were equivalent across all three groups. Moreover, we conducted sensitivity analyses controlling for both depression and anxiety (Supplementary Table 4), which confirmed that neither behavioural nor functional-connectivity results were altered by these comorbidities. Thus, neural-connectivity differences in the FP group appear robust to variation in affective symptoms.

The desired sample size for the FP group was not achieved due to challenges in recruitment. However, the target sample sizes for the SCI and HC groups were successfully met. To ensure the robustness of our findings and facilitate generalization, effect sizes were included in all analyses.

It remains challenging to disentangle the neural activity stemming from the underlying disorder processes from that generated by the symptom itself. Hence, it is important for future studies to include control groups with comparable symptom presentations. This approach would help clarify whether observed brain activity patterns are truly tied to the pathophysiology of FND or instead reflect broader, symptom-related effects.

## Supplementary Material

fcaf377_Supplementary_Data

## Data Availability

The data underpinning the results of this study are accessible from the corresponding author upon reasonable request. The analysis code used in this study is provided in the Supplementary material.
