# Supplementary material for: Revisiting motor inhibition in individuals with functional paralysis and spinal cord injury
Source: Brain Commun. 2025 Sep 27;7(5):fcaf377. doi: 10.1093/braincomms/fcaf377 (PMC12531627; doi:10.1093/braincomms/fcaf377)
Supplement: fcaf377_Supplementary_Data [file fcaf377_supplementary_data.docx]

**Supplementary material**

**Sample**

The following parameters were used to calculate the sample size: Based on previous magnetic resonance (MR) studies conducted by our team, a power of 0.8 was estimated with an expected effect size of 0.37, and the significance level (alpha) was set at 0.05. For an analysis of covariance (ANCOVA) comparing three independent groups, a total sample size of 74 participants was calculated, which results in approximately 25 participants per group. Due to recruitment difficulties, the target sample size was not quite reached for the functional paralysis (FP) group but was reached for the spinal cord injury (SCI) group and the healthy controls (HC).

**Acquisition parameters**

The anatomic T1-weighted images were acquired using a magnetization-prepared rapid gradient-echo (MPRAGE) sequence with the following parameters: repetition time (TR) / echo time (TE) of 8.3 ms / 3.9 ms, inversion time (TI) = 950 ms, turbo field echo (TFE) factor = 128, flip angle of 8°, slice thickness of 1 mm, field of view (FoV) of 256 × 256 × 180 mm³, voxel size of 1 × 1 × 1 mm³, and bandwidth of 191 Hz. For the functional T2-weighted echo-planar images, the following parameters were used: TR / TE of 2700 ms / 27 ms, flip angle of 80°, voxel size of 3 × 3 × 3 mm³, bandwidth of 2119 Hz, FoV of 240 × 240 × 159 mm³, and 356 repetitions.

**Normality test**

Normality of the data was assessed using the Shapiro–Wilk test for both response times (W = 0.95, p = 0.013) and accuracy (W = 0.56, p < 0.001), indicating that these data deviate significantly from a normal distribution.

**Pre-processing and denoising**

The realignment of the functional data was conducted with the SPM realign and unwarp procedure^1^. All scans were realigned to the first scan of the session using a least squares approach and a 6-parameter (rigid body) transformation^2^. To correct for motion and magnetic susceptibility interactions, the scans were resampled using b-spline interpolation. Potential outlier scans were identified using artifact detection tools^3^. The mean functional image was co-registered to each subject’s T1-weighted anatomical scan during normalization. The functional and anatomical images were normalized into standard MNI space and segmented into gray matter, white matter, and cerebrospinal fluid (CSF). Subsequently, the images were resampled into 2 mm isotropic voxels^4,5^ following a direct normalization procedure^6^. Finally, the functional images were smoothed using spatial convolution with a Gaussian kernel of 6 mm full width at half maximum.

The following confounders were denoised from the functional data^7,8^: white matter and CSF timeseries, motion parameters and their first-order derivatives (12 factors) to account for subject movement during the scan, outlier scans, session and task effects and their first-order derivatives (4 factors) to control for variability due to different scanning sessions, and cubic trends (4 factors) to account for low-frequency drifts within each functional run. Simultaneous high-pass frequency filtering of the BOLD timeseries above 0.01 Hz was applied.

**Characteristics**

Supplementary Table 1. Demographics and clinical characteristics

| **Variable** | | **Median (IQR)** | | | **Test statistics** | **P-value** |
| --- | --- | --- | --- | --- | --- | --- |
|  |  | **FP-group (n= 16)** | **SCI-group (n = 24)** | **Healthy controls (n=29)** |  |  |
| Age [in years] | | 37.5 (18.8) | 41 (16.2) | 33 (18) | χ2 = 4.28 | 0.118 |
| Sex [n of men] | | 6 | 18 | 8 | n.a. | 0.002 |
| Education [n] | |  |  |  | n.a. | 0.024 |
|  | Compulsory education | 2 | 2 | 2 |  |  |
|  | Upper secondary education | 2 | 0 | 4 |  |  |
|  | Vocational training/apprenticeship | 7 | 5 | 4 |  |  |
|  | Post-secondary non-tertiary education | 4 | 6 | 4 |  |  |
|  | University/college | 1 | 11 | 15 |  |  |
| Handedness [n of right-handed] | | 14 | 19 | 29 | n.a. | 0.022 |
| Analgesics [n] | | 5 | 4 | 1 | n.a. | 0.036 |
| Psychotropics [n] | | 6 | 3 | 3 | n.a. | 0.081 |
| Hospital Anxiety and Depression Scale (range: 0 - 21) | | | | |  |  |
|  | Total score depression | 4 (7) | 2.5 (4) | 2 (3) | χ2 = 10.42 | 0.005 |
|  | Total score anxiety | 7 (7) | 3.5 (4.25) | 4 (3) | χ2 = 10.39 | 0.006 |
| Satisfaction with Life Scale (range: 5 - 35) | | 23 (13.8) | 28.5 (6.25) | 30 (3) | χ2 = 10.81 | 0.004 |
| Numeric Rating Scale (range: 0 - 10) | | | | |  |  |
|  | Pain experienced now | 3 (6.25) | 0 (3) | 0 (1) | χ2 = 13.09 | 0.001 |
|  | Pain experienced in the past 7 days | 5 (4.5) | 1 (3.25) | 0 (1) | χ2 = 18.30 | < 0.001 |
| Time since symptom onset [years] | | 2.5 (3.5) | 11 (18.5) | n.a. | χ2 = 9.48 | 0.002 |
| Spinal Cord Independence Measure (range: 0 - 100) | | 88.5 (24.8) | 75 (27) | n.a. | χ2 = 2.49 | 0.114 |
| ISNCSCI | | | | |  |  |
|  | Total score motor (range: 0 - 100) | 85 (31) | 69 (46) | n.a. | χ2 = 1.57 | 0.21 |
|  | Total score light touch (range: 0 - 112) | 18.5 (20.8) | 13.5 (13.8) | n.a. | χ2 = 0.71 | 0.399 |
|  | Total score pinprick (range: 0 - 112) | 9 (16.2) | 15.5 (13.8) | n.a. | χ2 = 1.26 | 0.262 |
| Abbreviations: FP, functional paralysis; SCI, spinal cord injury; n.a., not applicable; ISNCSCI, International Standards for Neurological Classification of Spinal Cord Injury | | | | | | |
| Note: Test statistics from the Kruskal-Wallis rank sum test are reported. For Fisher's exact test, test statistics are not applicable. A p-value of less than 0.05 was considered significant. | | | | | | |

| Supplementary Table 2. Post hoc comparisons of demographic and clinical characteristics | | | |
| --- | --- | --- | --- |
| Variables | HC vs FP | FP vs SCI | HC vs SCI |
| Sex | p_FWE_ = 1.000 | p_FWE_ = 0.074 | p_FWE_ = 0.003 |
| Education | p_FWE_ = 0.037 | p_FWE_ = 0.082 | p_FWE_ = 1.000 |
| Handedness | p_FWE_ = 0.364 | p_FWE_ = 1.000 | p_FWE_ = 0.044 |
| Analgesics | p_FWE_ = 0.049 | p_FWE_ = 1.000 | p_FWE_ = 0.491 |
| HADS-D | W = 106.5 p_FWE_ = 0.008 | W = 252.5 p_FWE_ = 0.280 | W = 242.5 p_FWE_ = 0.170 |
| HADS-A | W = 115 p_FWE_ = 0.0161 | W = 298.5 p_FWE_ = 0.010 | W = 371 p_FWE_ = 1.000 |
| SWLS | W = 367.5 p_FWE_ = 0.004 | W = 119.5 p_FWE_ = 0.139 | W = 429 p_FWE_ = 0.445 |
| Abbreviations: HC, healthy controls; FP, functional paralysis group; SCI, spinal cord injury group; FWE, family-wise error rate; HADS-D, Hospital Anxiety and Depression Scale - Depression; HADS-A, Hospital Anxiety and Depression Scale - Anxiety; SWLS, Satisfaction with Life Scale.  Note: Group sizes were HC = 29, FP = 16, SCI = 24. Post-hoc pairwise comparisons were carried out with Fisher’s exact test for categorical variables (sex, education, handedness) and Wilcoxon rank-sum test for ordinal/score variables (HADS-D, HADS-A, SWLS). All p-values are two-tailed and Bonferroni-adjusted for family-wise error. “W” denotes the Wilcoxon rank-sum statistic. | | | |

**Behavioral Results**

Further accuracy analyses were conducted to examine performance differences between trial types and groups. Levene’s test for homogeneity of variance revealed a significant difference in variances in overall accuracy across groups (F_(2, 66)_ = 4.62, p = 0.013). For detailed condition-level analysis, we computed two additional accuracy measures: (1) go trial accuracy (hit rate), calculated as hits/(hits + misses) × 100, and (2) no-go trial accuracy (correct rejection rate), calculated as correct rejections/(correct rejections + false alarms) × 100. Kruskal-Wallis tests comparing these measures across groups showed no significant differences for either go trials (χ²(2) = 2.09, p = 0.352) or no-go trials (χ²(2) = 3.75, p = 0.154).

To test variability in response times across groups, we calculated each participant’s coefficient of variation (CV) and compared them using a Kruskal–Wallis test. The result indicated no significant difference between groups (χ2 = 4.07, p = 0.131).


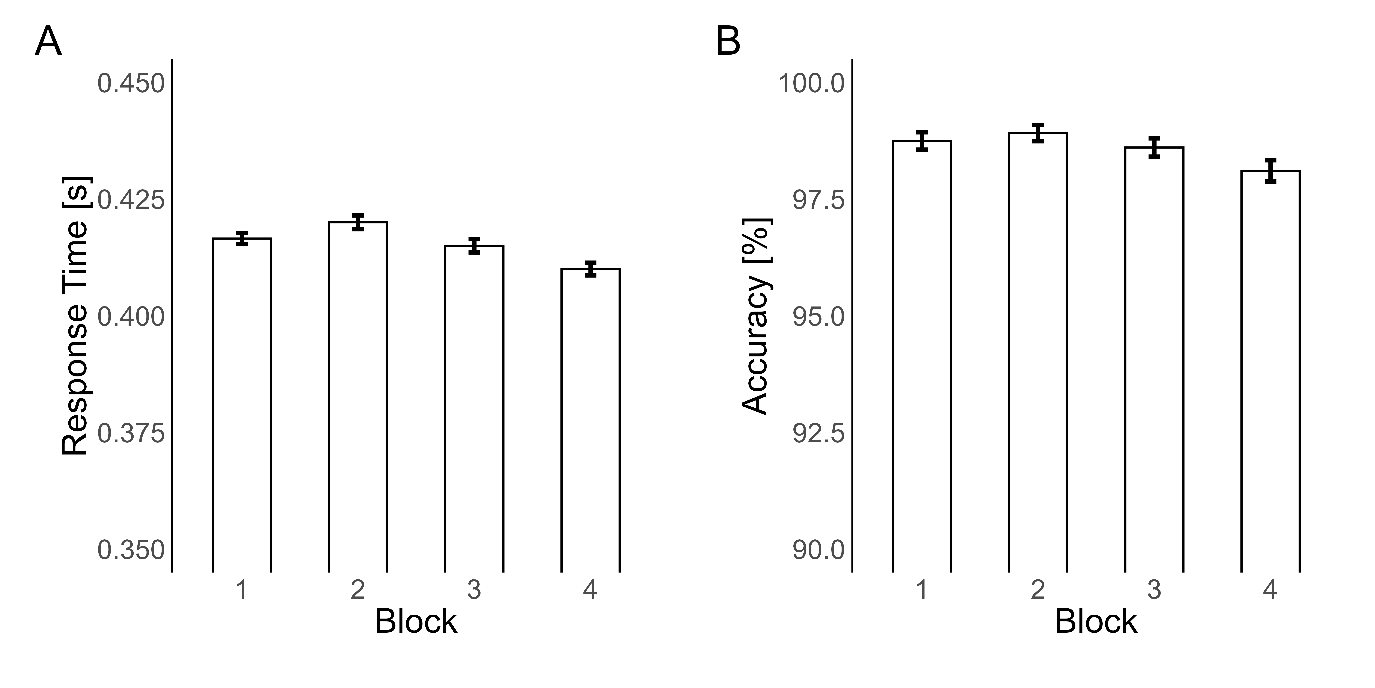


**Supplementary Figure 1. The stability of behavioral performance was assessed throughout the go/no-go task.**

(A) The average response time for each block is plotted for the entire sample (n = 69). Each bar represents the mean response time for a given block, with error bars showing the standard error of the mean (SEM). The y-axis has been zoomed in for visibility. The average response time shows a maximum change of 10 ms across task blocks.

(B) The accuracy shows a maximum change of 0.8% across task blocks for the entire sample (n = 69). Error bars represent the standard error of the mean (SEM). The y-axis has been zoomed in for visibility.

**Voxel-wise Validation of Motor Inhibition BOLD Activity**

The validation analysis showed activation of the motor inhibitory network, including regions such as the bilateral precentral gyrus, bilateral supplementary motor area (SMA), left superior parietal lobule, bilateral supramarginal gyrus, mid-cingulate cortex, right inferior frontal gyrus (triangular and opercular parts), right middle frontal gyrus, right angular gyrus, and left inferior occipital gyrus^9^. Sensorimotor areas, including cerebellar lobules 4, 5, 6, 8, and vermis 6 and 8, were also active^10^, alongside cortical regions such as the bilateral posterior cingulate gyrus, paracentral lobule, and inferior parietal lobule. Subcortical activation included the thalamic nuclei and left caudate. Further regions, including the bilateral precuneus, left rolandic operculum, left insula, bilateral superior frontal gyrus, bilateral inferior parietal lobule, and occipital and temporal areas, demonstrated significant activation (t = 4.88, p_FWE_ < 0.05, k > 20 voxels; see Supplementary Fig. 2 for motor inhibition and Supplementary Fig. 3 for the separate effects of hit and correct rejection).


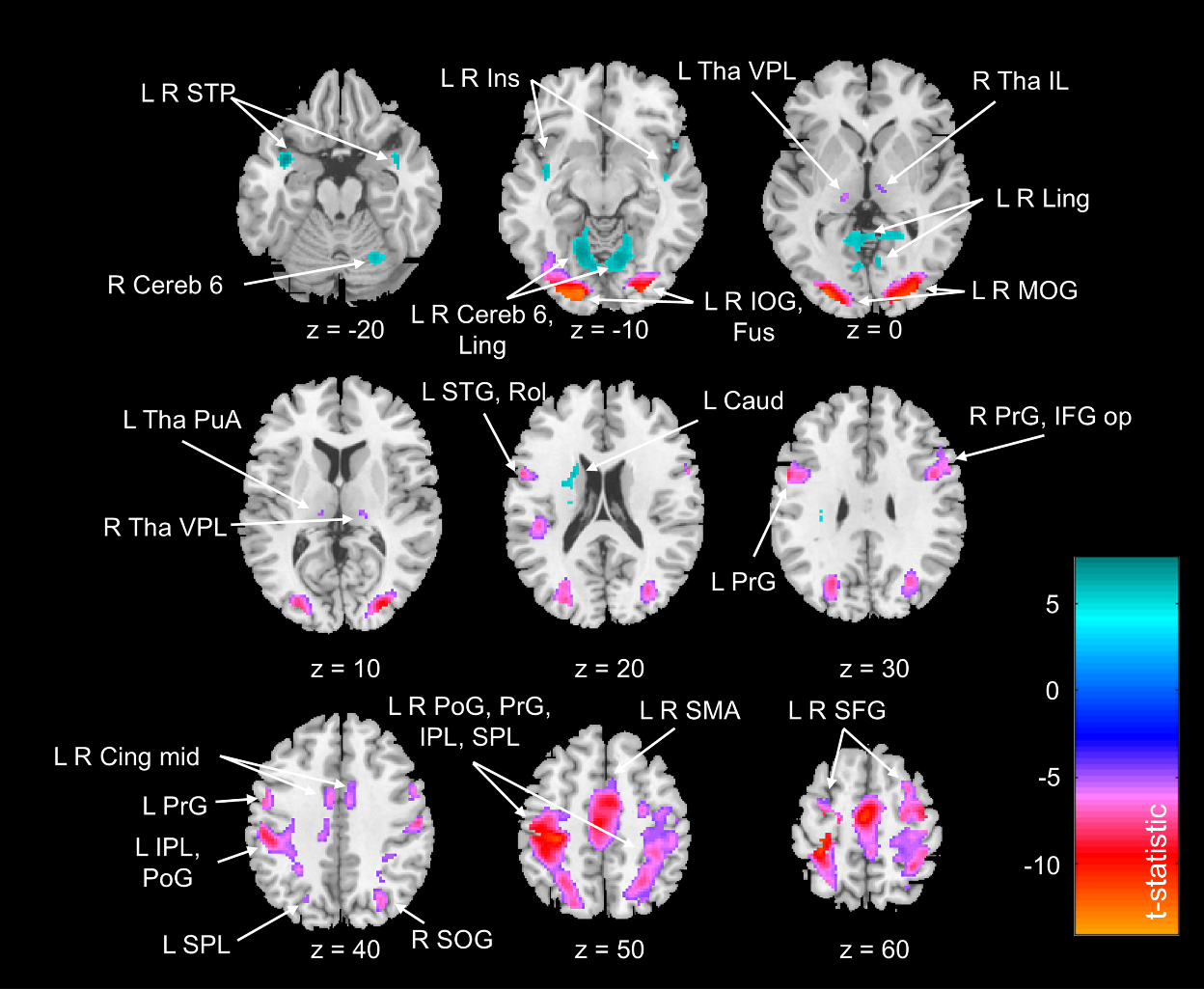


**Supplementary Figure 2.** Group-level activation maps for motor inhibition (positive t-values) and execution (negative t-values) during the go/no-go task (n = 69). The color bar represents t-statistic magnitudes. Maps are thresholded at cluster-level family-wise error-corrected p < 0.05 (extent threshold: k = 20 voxels; height threshold: t = 4.88).

Abbreviations: STP, superior temporal pole; Cereb, cerebellum; Ins, insula; Ling, lingual gyrus; IOG, inferior occipital gyrus; Fus, fusiform gyrus; Tha VPL, thalamus ventral posterolateral nucleus; Tha IL, thalamus intralaminar nuclei; MOG, middle occipital gyrus; Tha PuA, thalamus anterior pulvinar nucleus; STG, superior temporal gyrus; Rol, rolandic operculum; Caud, caudate; PrG, precentral gyrus; IFG op, inferior frontal gyrus opercular part; Cing mid, mid-cingulate cortex; IPL, inferior parietal lobule; PoG, postcentral gyrus; SPL, superior parietal lobule; SOG, superior occipital gyrus; SMA, supplementary motor area; SFG, superior frontal gyrus.


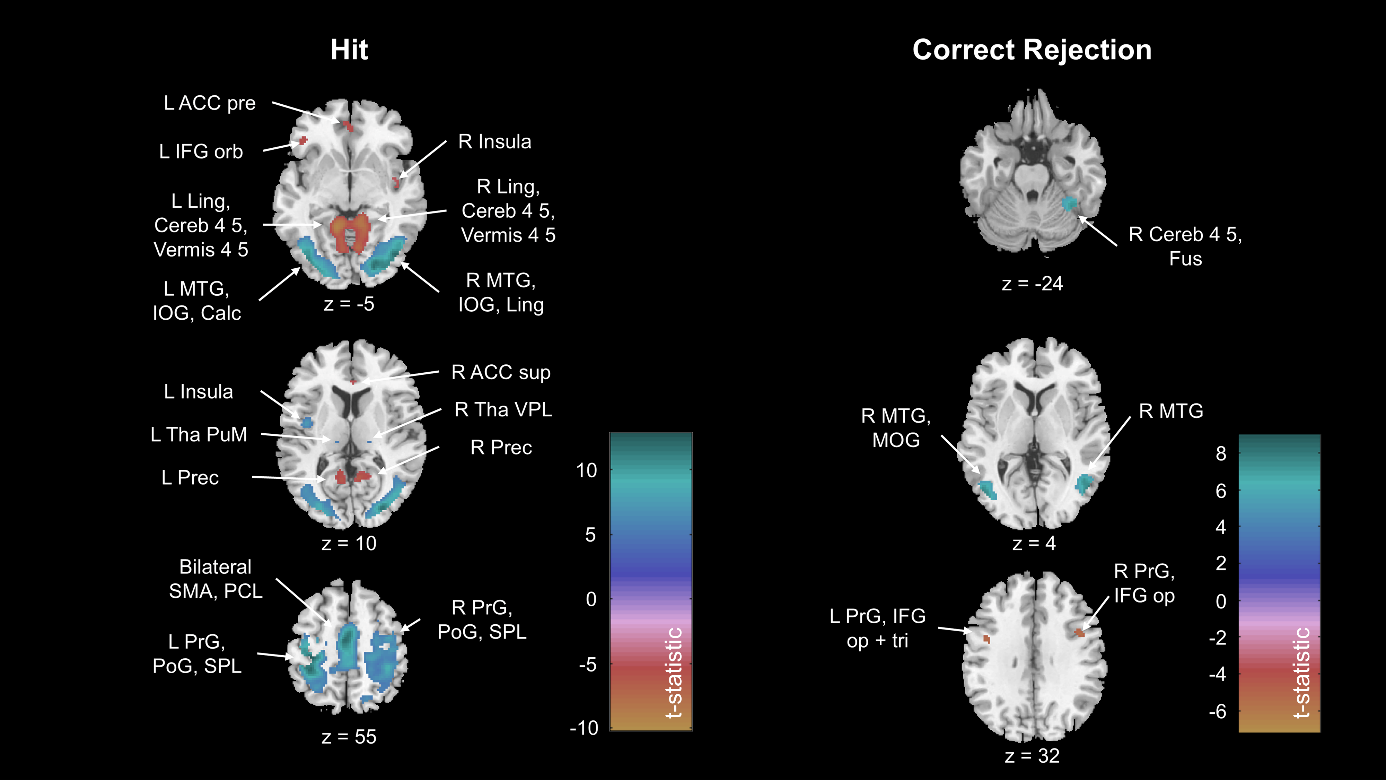
**Supplementary Figure 3.** Results (n = 69) of the control analysis for only hit (left) and only correct rejection (right). The color bars represent t-statistic magnitudes. Activation maps are thresholded at a cluster-level family-wise error corrected p < 0.05, with an extent threshold of k = 20 voxels. The height threshold for significance is set at t = 4.88.

Abbreviations: ACC pre, pregenual anterior cingulate cortex; IFG orb, inferior frontal gyrus orbital part; Ling, lingual gyrus; Cereb, cerebellum; MTG, middle temporal gyrus; IOG, inferior occipital gyrus; Calc, calcarine cortex; Tha PuM, thalamus medial pulvinar nucleus; Prec, precuneus; ACC sup, supracallosal anterior cingulate cortex; Tha VPL, thalamus ventral posterolateral nucleus; SMA, supplementary motor area; PCL, paracentral lobule; PrG, precentral gyrus; PoG, postcentral gyrus; SPL, superior parietal lobule; Fus, fusiform gyrus; MOG, middle occipital gyrus; IFG op, inferior frontal gyrus opercular part; IFG tri, inferior frontal gyrus triangular part.

| Supplementary Table 3. Significant Group Differences in BOLD Activity During Correct Rejection vs. Hit | | | | | |
| --- | --- | --- | --- | --- | --- |
| Contrast | Region | MNI | k (voxels) | p-FWE | statistic |
| FP > HC | Left insula, left superior temporal gyrus | -44 0 -4 | 146 | 0.02 | 5.02 |
| FP > HC | Left posterior cingulate cortex, bilateral precuneus | -6 -46 16 | 165 | 0.011 | 4.34 |
| HC > FP | Left postcentral gyrus, left inferior parietal lobule | -30 -26 48 | 176 | 0.008 | 4.23 |
| SCI > FP | Right postcentral gyrus, right precentral gyrus | 46 -18 38 | 154 | 0.016 | 5.15 |
| SCI > HC | Right postcentral gyrus, right precentral gyrus | 42 -20 38 | 346 | < 0.001 | 5.19 |
| SCI > HC | Bilateral precuneus | 0 -70 42 | 119 | 0.045 | 4.3 |
| Abbreviations: FP, functional paralysis group; HC, healthy controls; SCI, spinal cord injury group; MNI, Montreal Neurological Institute standard space coordinates; k, cluster size; FWE, Family-wise error-corrected. Note: All contrasts were thresholded at p < 0.001 (uncorrected) at the voxel level, with only clusters surviving p < 0.05 FWE-corrected at the cluster level reported. Only significant comparisons are shown. BOLD activity reflects differences during correct rejection trials vs. hit trials. | | | | | |

**Sensitivity Analyses**

| Supplementary Table 4. Sensitivity Analyses: Behavioral and ROI-Level Functional Connectivity Results | | | |
| --- | --- | --- | --- |
| Model | Partial η² (group) | statistic (df) | p-value |
| Response Time (Hits) | | | |
| RT ~ group | 0.04 | χ2 = 2.71 _(2)_ | 0.258 |
| RT ~ group + age + handedness + sex + HADS-D + HADS-A + analgesics + psychotropics | 0.001 | χ2 = 0.07 _(2)_ | 0.968 |
| Response Time (False Alarms) | | | |
| RT ~ group | 0.12 | χ2 = 5.74 _(2)_ | 0.057 |
| RT ~ group + age + handedness + sex + HADS-D + HADS-A + analgesics + psychotropics | 0.01 | χ2 = 0.46 _(2)_ | 0.793 |
| Accuracy | | | |
| ACC ~ group | 0.06 | χ2 = 4.33 _(2)_ | 0.115 |
| ACC ~ group + age + handedness + sex + HADS-D + HADS-A + analgesics + psychotropics | 0.02 | χ2 = 1.52 _(2)_ | 0.467 |
| Cluster 1 | | | |
| FC ~ group + age + handedness | 0.34; CI 95% [0.18, 1.00] | F = 17.59 _(2, 64)_ | < 0.001 |
| FC ~ group + age + handedness + sex + HADS-D + HADS-A + analgesics + psychotropics | 0.36; CI 95% [0.19, 1.00] | F = 10.22 _(2, 59)_ | < 0.001 |
| Cluster 2 | | | |
| FC ~ group + age + handedness | 0.34; CI 95% [0.18, 1.00] | F = 15.31 _(2, 64)_ | < 0.001 |
| FC ~ group + age + handedness + sex + HADS-D + HADS-A + analgesics + psychotropics | 0.34; CI 95% [0.18, 1.00] | F = 14.13 _(2, 59)_ | < 0.001 |
| Abbreviations: RT, response time; ACC, accuracy; FC, functional connectivity; HADS-A, Hospital Anxiety and Depression Scale – Anxiety; HADS-D, Hospital Anxiety and Depression Scale – Depression; η², partial eta squared; CI, confidence interval; df, degrees of freedom. Note: Behavioral models: Group effects were first assessed using Kruskal-Wallis tests (χ²). Covariate-adjusted models were then fitted by regressing out effects of age, handedness, sex, HADS scores, and medication (analgesics/psychotropics) from residuals before testing group effects. Functional connectivity models: For each cluster (cluster 1: right precentral gyrus [seed] and left insula; cluster 2: right precentral gyrus [seed], left medial superior frontal gyrus, and bilateral supplementary motor area), two models were fitted: (1) a baseline model adjusted for age and handedness, and (2) a sensitivity model further adjusted for sex, HADS-Anxiety, and HADS-Depression to evaluate robustness. The group effect was assessed via partial η² and Type III ANOVA p-values; models were compared using likelihood ratio tests, and coefficients were reported with 95% CIs. | | | |

**Analysis Code**

# go_no_go_analysis.R

# Task-based fMRI analysis for Go/No‑Go data

# --------------------------------------------------

# 1. Load packages

library(tidyverse)

library(lme4)

library(car)

library(ggplot2)

library(ggpubr)

# 2. Set working directory to script location (requires RStudio)

setwd(dirname(rstudioapi::getSourceEditorContext()$path))

# 3. Read & preprocess data

go_nogo <- read_csv('go_nogo.csv') %>%

    mutate(condition = factor(condition),

         group     = factor(group))

# Split into hits and false alarms

hit <- filter(go_nogo, condition == 'hit')

FA  <- filter(go_nogo, condition == 'false_al')

# 4. Exclude extreme RTs (±2 SD) and non‑positive values

summarize_rt <- function(df) {

  m <- mean(df$key_resp.rt, na.rm = TRUE)

  s <- sd(df$key_resp.rt, na.rm = TRUE)

  df %>%

    filter(between(key_resp.rt, m - 2*s, m + 2*s),

           key_resp.rt > 0)

}

hit_filt <- summarize_rt(hit)

FA_filt  <- summarize_rt(FA)

# 5. Test RT differences across groups

# 5a. Descriptives by group

hit_filt %>% group_by(group) %>% summarize(mean_rt = mean(key_resp.rt),

                                           sd_rt   = sd(key_resp.rt))

# 5b. Levene’s test for homogeneity

leveneTest(key_resp.rt ~ group, data = hit_filt)

# 5c. Participant‑level means and nonparametric test

participant_rt <- hit_filt %>%

  group_by(participant, group, age) %>%

  summarize(mean_rt = mean(key_resp.rt), .groups = 'drop')

kruskal.test(mean_rt ~ group, data = participant_rt)

# 5d. Effect size (generalized η²) for Kruskal–Wallis

compute_eta2G <- function(ranks, groups) {

  grp_sizes  <- table(groups)

  grp_ranks  <- tapply(ranks, groups, sum)

  total_r    <- sum(ranks)

  mean_r     <- total_r / length(ranks)

  ss_eff     <- sum((grp_ranks^2)/grp_sizes) - total_r^2/length(ranks)

  ss_tot     <- sum((ranks - mean_r)^2)

  ss_err     <- ss_tot - ss_eff

  ss_eff/(ss_eff + ss_err)

}

eta2G_rt <- compute_eta2G(rank(participant_rt$mean_rt), participant_rt$group)

cat("RT η²G =", round(eta2G_rt, 3), "\n")

# 6. Sensitivity analysis with covariates

demog <- read_csv2('task_fMRI_FND_demog.csv') %>%

  slice(-c(6, 31)) %>%

  mutate(group = factor(group))

hand  <- read_csv('hand.csv')  %>% slice(-c(6, 31))

hadsa <- read_csv('HADS-A.csv') %>% slice(-c(6, 31))

hadsd <- read_csv('HADS-D.csv') %>% slice(-c(6, 31))

meds  <- read_csv('meds.csv')  %>% slice(-c(6, 31))

RT_cov <- participant_rt %>%

  left_join(demog, by = c("participant")) %>%

  bind_cols(hand, hadsa, hadsd, meds, demog)

# Remove covariate effects and rerun KW

RT_cov$rt_resid <- resid(lm(mean_rt ~ age + sex_female + hand +

                              hadsa + hadsd + analgesics + psychotropic,

                            data = RT_cov))

kw_resid <- kruskal.test(rt_resid ~ group, data = RT_cov)

cat("Residual RT KW p-value =", kw_resid$p.value, "\n")

# 7. Response‑time plots

# Boxplot of hit RT by group

ggplot(participant_rt, aes(group, mean_rt)) +

  geom_boxplot(outlier.shape = NA, width = 0.6) +

  geom_jitter(width = .1, alpha = .7) +

  labs(x = NULL, y = "Mean RT (s)") +

  theme_minimal()

# RT time series (all trials)

ggplot(go_nogo, aes(trial_start, key_resp.rt)) +

  geom_line(alpha = .6) +

  geom_smooth(method = "loess") +

  labs(x = "Time (s)", y = "RT (s)") +

  theme_minimal()

# 8. Accuracy analyses

accuracy <- go_nogo %>%

  group_by(participant, group, age) %>%

  summarize(

    overall_acc = mean(key_resp.corr, na.rm = TRUE) * 100,

    hit_acc     = mean(condition == "hit",    na.rm = TRUE) * 100,

    rej_acc     = mean(condition == "corr_rej", na.rm = TRUE) * 100,

    .groups = 'drop'

  )

# Nonparametric tests

kruskal.test(overall_acc ~ group, data = accuracy)

# Effect size

eta2G_acc <- compute_eta2G(rank(accuracy$overall_acc), accuracy$group)

cat("Accuracy η²G =", round(eta2G_acc, 3), "\n")

#sensitivity analysis

ACC <- cbind(accuracy, demog, hand, hadsa, hadsd, meds)

ACC$acc_resid <- resid(lm(overall_acc~age+sex_female+hand+hadsa+hadsd+analgesics+psychotropic, data=ACC))

kw_acc <- kruskal.test(acc_resid~group, data=ACC)

print(kw_acc)

rks <- rank(ACC$acc_resid); gs <- table(ACC$group); gr <- tapply(rks, ACC$group, sum)

tot <- sum(rks); m_r <- tot/nrow(ACC)

ss_e <- sum((gr^2)/gs) - tot^2/nrow(ACC)

ss_t <- sum((ACC$acc_resid - m_r)^2); ss_err <- ss_t-ss_e

eta2G_acc <- ss_e/(ss_e+ss_err)

cat("Acc cov-adjusted η²G=", round(eta2G_acc,3),"\n")

# Accuracy boxplot

ggplot(accuracy, aes(group, overall_acc)) +

  geom_boxplot(outlier.shape = NA, width = 0.6) +

  geom_jitter(width = .1, alpha = .7) +

  labs(x = NULL, y = "Accuracy (%)") +

  theme_minimal()

# 9. Accuracy over blocks

block_summary <- go_nogo %>%

  group_by(block_loop.thisN) %>%

  summarize(

    mean_acc = mean(key_resp.corr, na.rm = TRUE) * 100,

    se_acc   = sd(key_resp.corr, na.rm = TRUE) / sqrt(n()) * 100

  )

ggplot(block_summary, aes(factor(block_loop.thisN), mean_acc)) +

  geom_col(fill = NA, color = "black") +

  geom_errorbar(aes(ymin = mean_acc - se_acc, ymax = mean_acc + se_acc),

                width = 0.2) +

  labs(x = "Block", y = "Accuracy (%)") +

  theme_minimal()

# task_fMRI_conn_analysis.R

# Streamlined analysis of significant FC clusters from CONN

# --------------------------------------------------------

# 1. Load packages

library(tidyverse)    # Data wrangling & ggplot2

library(rstatix)      # Statistical tests

library(ggpubr)       # ggplot enhancements

library(broom)        # Tidy model output

library(emmeans)      # Estimated marginal means

library(car)          # Anova type III

library(effectsize)   # Effect size calculations

library(boot)         # Bootstrap

# 2. Set working directory

setwd(dirname(rstudioapi::getSourceEditorContext()$path))

# 3. Read & merge data

demog   <- read_csv2('task_fMRI_FND_demog.csv') %>%

  mutate(ID = row_number(), group = factor(group))

clus    <- read_csv('aal_2_clus.csv')

hand    <- read_csv('hand.csv')

hadsa   <- read_csv('HADS-A.csv')

hadsd   <- read_csv('HADS-D.csv')

meds    <- read_csv('meds.csv')

tsi     <- read_csv('tsi.csv')

data <- clus %>%

  bind_cols(demog, hand, hadsa, hadsd, meds, tsi) %>%

  drop_na()

# 4. Medications: Fisher tests by group

# Specify which meds you want to test

meds <- c("psychotropic", "analgesics")

res <- tibble(med = meds) %>%

  rowwise() %>%

  mutate(

    # build the contingency table: rows = 0/1, cols = group

    tbl = list(table(factor(data[[med]], levels = c(0,1)), data$group)),

    # run Fisher's exact test on the 2×3 table

    p_value = fisher.test(tbl)$p.value

  ) %>%

  ungroup() %>%

  # Bonferroni‐correct the two p‐values

  mutate(p_adj = p.adjust(p_value, method = "bonferroni"))

print(res)

# 5. Recode factors & define contrasts

data <- data %>%

  mutate(

    group       = factor(group, levels=c('FND','SCI','HC')),

    hand        = factor(hand),

    psychotropic= factor(psychotropic),

    analgesics  = factor(analgesics),

    SMA_ch = SMA_PrG_corr_rej - SMA_PrG_hit,

    Ins_ch = Ins_PrG_corr_rej - Ins_PrG_hit

  )

# 6. ANCOVA helper function

run_ancova <- function(df, response) {

  formula <- as.formula(paste(response, '~ age + hand + group'))

  lm_fit  <- lm(formula, data=df)

  aov_res <- Anova(lm_fit, type='III')

  eta     <- eta_squared(lm_fit, partial=TRUE)

  emm     <- emmeans(lm_fit, pairwise~group, adjust='bonferroni')

  list(

    lm      = lm_fit,

    ancova  = aov_res,

    eta     = eta,

    emmeans = emm

  )

}

# 7. Analyze SMA contrast

sma_res <- run_ancova(data, 'SMA_ch')

print(sma_res$ancova)

print(sma_res$eta)

print(sma_res$emmeans)

# 8. Plot SMA results

p_sma <- ggplot(data, aes(group, SMA_ch)) +

  geom_boxplot() +

  geom_jitter(width=0.1, alpha=0.7) +

  stat_pvalue_manual(

    broom::tidy(sma_res$emmeans$contrasts) %>%

      mutate(

        y.position = max(data$SMA_ch) + seq(0.02, by=0.02, length.out=n()),

        label = case_when(

          p.value<.001 ~ '***', p.value<.01 ~ '**', p.value<.05 ~ '*', TRUE~'ns'

        )

      ),

    label='label', tip.length=0, size=5

  ) +

  labs(x=NULL, y='Δ FC (SMA)') + theme_minimal()

# 9. Analyze Insula contrast

ins_res <- run_ancova(data, 'Ins_ch')

print(ins_res$ancova)

print(ins_res$eta)

print(ins_res$emmeans)

# 10. Plot Insula results

p_ins <- ggplot(data, aes(group, Ins_ch)) +

  geom_boxplot() +

  geom_jitter(width=0.1, alpha=0.7) +

  stat_pvalue_manual(

    broom::tidy(ins_res$emmeans$contrasts) %>%

      mutate(

        y.position = max(data$Ins_ch) + seq(0.02, by=0.02, length.out=n()),

        label = case_when(

          p.value<.001 ~ '***', p.value<.01 ~ '**', p.value<.05 ~ '*', TRUE~'ns'

        )

      ),

    label='label', tip.length=0, size=5

  ) +

  labs(x=NULL, y='Δ FC (Insula)') + theme_minimal()

# Display plots

print(p_sma)

print(p_ins)

# 11. Sensitivity analysis: add covariates

model_full <- lm(SMA_ch ~ group + age + hand + sex_female + `HADS-A` + `HADS-D` + analgesics + psychotropic,

                 data=data)

eta_orig <- eta_squared(sma_res$lm, partial=TRUE) %>% filter(Parameter=='group')

eta_full <- eta_squared(model_full, partial=TRUE) %>% filter(Parameter=='group')

ANOVA_comparison <- anova(sma_res$lm, model_full)

list(eta_orig=eta_orig, eta_full=eta_full, model_comp=ANOVA_comparison)

# 12 Spearman correlations with clinical scores

scores <- data %>% filter(group!='HC')

# TSI vs SMA, Ins

scores %>%

  group_by(group) %>%

  summarise(

    rho_sma = cor(tsi, SMA_ch, method='spearman'),

    p_sma   = cor.test(tsi,SMA_ch,method='spearman')$p.value,

    rho_ins = cor(tsi, Ins_ch, method='spearman'),

    p_ins   = cor.test(tsi,Ins_ch,method='spearman')$p.value

  )

# 13. Correlation vs accuracy

go_nogo <- read_csv('go_nogo.csv') %>%

  group_by(participant, group) %>%

  summarise(

    acc = mean(key_resp.corr)*100,

    rt  = mean(key_resp.rt)

  )

merged  <- inner_join(data, go_nogo, by=c('ID'='participant','group'))

cor.test(merged$Ins_ch, merged$acc, method='spearman')

#Bootstrap Spearman correlation (Ins_ch vs. accuracy)

library(boot)

# 1) Define a function that returns Spearman’s ρ for a resampled dataset

cor_fun <- function(dat, indices) {

  d <- dat[indices, ]

  cor(d$Ins_ch, d$acc, method = "spearman")

}

# 2) Run the bootstrap (10 000 replications)

set.seed(2025)  # for reproducibility

boot_res <- boot(data = merged, statistic = cor_fun, R = 10000)

# 3) Print the raw bootstrap results

print(boot_res)

# 4) Compute bias‑corrected & accelerated confidence intervals

boot_ci <- boot.ci(boot_res, type = "bca")

print(boot_ci)

# 14. Final combined plot: Ins_ch vs accuracy

ggplot(merged, aes(acc, Ins_ch, color=group)) +

  geom_point() + geom_smooth(method='lm', se=TRUE) +

  labs(x='Accuracy [%]', y='FC (Insula)') +

  theme_minimal(base_size=14)

**References**

1. Andersson JLR, Hutton C, Ashburner J, Turner R, Friston K. Modeling Geometric Deformations in EPI Time Series. *NeuroImage*. 2001;13(5):903-919. doi:10.1006/nimg.2001.0746

2. Friston KJ, Ashburner J, Frith CD, Poline JB, Heather JD, Frackowiak RSJ. Spatial registration and normalization of images. *Human Brain Mapping*. 1995;3(3):165-189. doi:10.1002/hbm.460030303

3. Whitfield-Gabrieli S, Nieto-Castanon A, Ghosh S. Artifact detection tools (ART). Published online 2011.

4. Ashburner J, Friston KJ. Unified segmentation. *NeuroImage*. 2005;26(3):839-851. doi:10.1016/j.neuroimage.2005.02.018

5. Ashburner J. A fast diffeomorphic image registration algorithm. *NeuroImage*. 2007;38(1):95-113. doi:10.1016/j.neuroimage.2007.07.007

6. Calhoun VD, Wager TD, Krishnan A, et al. The impact of T1 versus EPI spatial normalization templates for fMRI data analyses. *Human Brain Mapping*. 2017;38(11):5331-5342. doi:10.1002/hbm.23737

7. Friston KJ, Williams S, Howard R, Frackowiak RSJ, Turner R. Movement-related effects in fMRI time-series. *Magnetic resonance in medicine*. 1996;35(3):346-355.

8. Behzadi Y, Restom K, Liau J, Liu TT. A component based noise correction method (CompCor) for BOLD and perfusion based fMRI. *NeuroImage*. 2007;37(1):90-101. doi:10.1016/j.neuroimage.2007.04.042

9. Zhang R, Geng X, Lee TMC. Large-scale functional neural network correlates of response inhibition: an fMRI meta-analysis. *Brain Struct Funct*. 2017;222(9):3973-3990. doi:10.1007/s00429-017-1443-x

10. Schmahmann JD. The cerebellum and cognition. *Neuroscience Letters*. 2019;688:62-75. doi:10.1016/j.neulet.2018.07.005
